# Bidirectional information sharing between Nagoya Memorial Hospital and health insurance pharmacies using a communication sheet for pharmaceutical cooperation

**DOI:** 10.1186/s40780-020-00177-5

**Published:** 2020-10-07

**Authors:** Megumi Kabeya, Satoshi Hibi, Shu Yuasa, Satoshi Kayukawa, Kenji Ina

**Affiliations:** 1 Department of Pharmacy, Nagoya Memorial Hospital, 4-305 Hirabari, Tenpaku-ku, Nagoya, 468-8520 Japan; 2 Department of Hematology, Nagoya Memorial Hospital, 4-305 Hirabari, Tenpaku-ku, Nagoya, 468-8520 Japan; 3 Department of Psychosomatic Medicine, Nagoya Memorial Hospital, 4-305 Hirabari, Tenpaku-ku, Nagoya, 468-8520 Japan

**Keywords:** Communication sheet, Cooperation with health insurance pharmacies, Bidirectional information sharing, Cancer, Outpatient pharmacy services

## Abstract

**Background:**

We collaborated with the regional pharmaceutical associations near Nagoya Memorial Hospital and created a communication sheet for pharmaceutical cooperation between the hospital and health insurance pharmacies.

**Methods:**

The communication sheet for pharmaceutical cooperation was issued in October 2014. We conducted a questionnaire survey of both cancer patients and community pharmacists 1 year after the implementation of the use of this sheet. Based on the results of the survey, we modified our communication sheet and added a unified reply form in October 2016. We examined the number of replies from community pharmacists from October 2014 to April 2019. We then analyzed how community pharmacists instructed and communicated with cancer patients using the results of both the questionnaire survey and the reply form, which were compared before and after introducing the modified version of the communication sheet.

**Results:**

During the 5 years of observation, 743 communication sheets were sent from Nagoya Memorial Hospital to community pharmacists. As a result of pharmaceutical cooperation in using the communication sheet, 96.4% of prescribed medication were immediately prepared in health insurance pharmacies on that day. The communication sheet also enhanced the conversations between cancer patients and pharmacists. The introduction of the unified reply form increased the response rate of community pharmacists from 1.7 to 69.5% (*p* < 0.001). The communication between community pharmacists and cancer patients was significantly hindered by prescriptions without an oral cancer drug and patient age < 65 years old (*p* < 0.05). However, this hindrance was reduced by the use of the modified form.

**Conclusions:**

The communication sheet for pharmaceutical cooperation is useful for bidirectional information sharing between hospitals and health insurance pharmacies, which may enable pharmacists to provide cancer patients with medication instructions in coordination with hospitals and increase the quality of outpatient pharmacy services.

## Background

Patients receiving antineoplastic agents can have complex medication requirements associated with the management of adverse effects and cancer pain [1]. Barriers to optimal use of medications among cancer patients may include the complexity of medication regimens or inadequate communication with medical staff members [2]. Moreover, adherence to medications often decreases with age and polypharmacy [3, 4], and non-adherence results in decreased survival [5] and increased recurrence rates in cancer patients [6]. The knowledge and skills of pharmacists may support a wide variety of functions to achieve optimal outcomes of cancer chemotherapy. Patient education by pharmacists should improve treatment adherence [7, 8], and comprehensive management of medication therapy is critical for cancer patients undergoing chemotherapy. Because many oncologists find it difficult to schedule sufficient time to counsel patients in clinical practice, pharmacists have a key role in bridging gaps between physicians and cancer patients [9, 10].

In Japan, the number of health insurance pharmacies has increased since the pharmaceutical law was amended in 1951 [11]. The sharing of external prescriptions was high in Nagoya Memorial Hospital, and currently, approximately 90% of cancer patients receive medications outside the hospital [12]. There are several problems in outpatient pharmacy services related to chemotherapy. First, cancer patients are sometimes prescribed expensive or rare medications and are unable to immediately receive them due to these medications being out of stock at a nearby pharmacy. Second, community pharmacists have difficulties supporting cancer patients, mainly because information obtained from externally dispensed prescriptions alone is far from sufficient to instruct individual patients. Especially in the treatment of malignancy, a variety of adverse events are caused by either chemotherapeutic agents or analgesics, including opioids, so frequently [13, 14]that timely management and intimate cooperation between health insurance pharmacies and hospitals is required [15, 16].

With the aim to enhance the quality of patient support, a questionnaire survey was administered to both cancer patients and community pharmacists in 2014 and demonstrated that all patients had a family pharmacy and that the patients receiving oral cancer medications should consult with community pharmacists more often than those receiving parenteral chemotherapeutic agents [17]. Only 10% of pharmacists often discussed cancer treatments with patients at the window, and almost all pharmacists were eager to obtain more concrete information on cancer patients from the hospitals. Based on the results of the survey, we created an original communication sheet and initiated pharmaceutical cooperation. The purpose of the present study was to identify points to be improved in our communication system between the hospital and community pharmacists.

## Methods

**Fig. 1:**
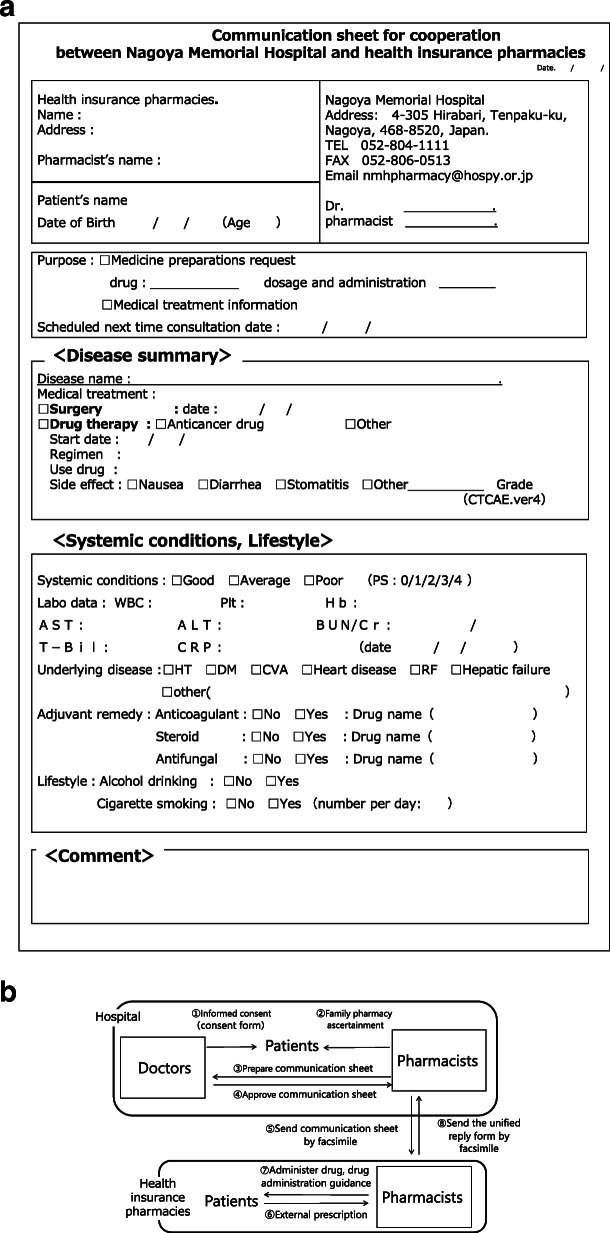
**a** Communication sheet for pharmaceutical cooperation between Nagoya Memorial Hospital and health insurance pharmacies. **b** Flow of pharmaceutical cooperation using the communication sheet

**Fig. 2:**
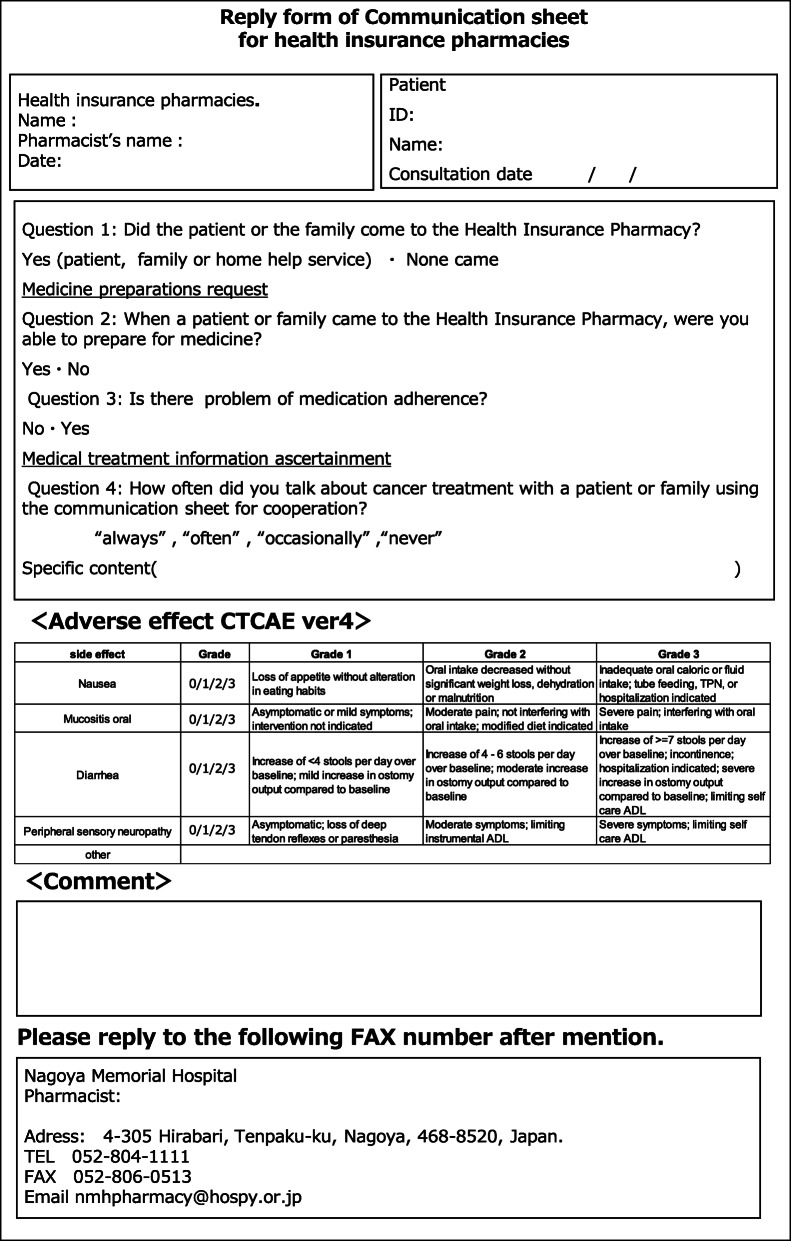
Unified reply form of the communication sheet

Pharmaceutical cooperation using the communication sheet was initiated for cancer patients in October 2014. The information sheet was sent from the hospital to the health insurance pharmacy by facsimile after obtaining informed consent from the patients (Fig. 1a and b). The following data were extracted from the communication sheet for pharmaceutical cooperation: the characteristics of cancer outpatients, cancer type, medical department issuing outpatient prescriptions, and responses from community pharmacists. One year after the implementation of this communication system, a second questionnaire survey comprising five questions was administered to both cancer patients and community pharmacists to evaluate the communication sheet for pharmaceutical cooperation. A total of 97 patients receiving chemotherapeutics answered the survey, which included the following questions: (1) Do you consult with a community pharmacist about the cancer treatments you receive? (2) Do you discuss your type of cancer with a community pharmacist? (3) Do you discuss the administration method of antineoplastic agents with a community pharmacist? (4) Do you consult with a community pharmacist about your treatments other than pharmacotherapy? (5) Do you receive an explanation about the adverse effects and the respective treatments at a nearby pharmacy? The attending community pharmacists who served the above patients also replied to the corresponding five questions: (1) Do you talk to cancer patients about the cancer treatments they receive? (2) Do you talk with cancer patients about their types of cancer? (3) Do you talk with cancer patients about their methods of administering their antineoplastic agents? (4) Do you talk with cancer patients about their treatments other than pharmacotherapy? (5) Do you instruct patients about adverse effects and the respective treatments? A three-point Likert scale was used: 1) I often talk from before. 2) I talk more than before. 3) I never talk. We then analyzed these results and examined the communication between cancer patients and community pharmacists. Based on the results of the second survey, the communication sheet for pharmaceutical cooperation was revised and a unified reply form from community pharmacists was added in October 2016 (Fig. 2). Each grade of related adverse effects was described in this form according to the Common Terminology Criteria for Adverse Events version 4.0 (CTCAE ver.4), to which community pharmacists can easily refer during the medication guidance for cancer patients. The findings were compared using the results of the survey and reply forms before and after the revision in October 2016. Free comments written by community pharmacists on the reply form were also examined.

The data analysis was primarily performed using descriptive statistics. Fisher’s exact test was used to compare the results of a stratified analysis. The communication barriers for patients or pharmacists are able to talk about cancer therapy in health insurance pharmacy was compared by applying Fisher’s exact test. Univariate analysis was performed using age, gender, clinical department, area of pharmacy, oral cancer drug prescription as independent variables. To identify risk factors associated with communication barrier, multiple logistic regression analysis was performed. Factors for which *P* < 0.20 in univariate analysis were selected for multiple logistic regression analysis. All *P*-values were two sided, and *P*-values of 0.05 or less were considered statistically significant. All statistical analyses were performed with EZR (Saitama Medical Center, Jichi Medical University, Saitama, Japan), which is a graphical user interface for R (The R Foundation for Statistical Computing, Vienna, Austria). More precisely, it is a modified version of R commander designed to add statistical functions frequently used in biostatistics [18]. The research protocol was prepared in compliance with ethical guidelines for epidemiological research. The protocol was approved by the Research Ethics Committee of Nagoya Memorial Hospital.

## Results

The number of communication sheets issued from Nagoya Memorial Hospital from October 2014 to April 2019 was 743. The clinical characteristics of cancer patients are shown in Table 1. The types of cancer included colorectal cancer (*N* = 199), lung cancer (*N* = 137), gastric cancer (*N* = 93), breast cancer (*N* = 53), lymphoma (*N* = 37), ovarian cancer (*N* = 26), leukemia (*N* = 21), and pancreatic cancer (*N* = 19). Among the medical departments that issued communication sheets for cancer patients, the first was the medical oncology/hematology department (62%), followed by the respiratory medicine (18%), gastrointestinal medicine (6%), gynecology (6%), orthopedics (3%), urology (2%), pediatrics (1%), and other (2%) departments. With the use of communication sheets for pharmaceutical cooperation, all cancer patients received externally prescribed drugs at a nearby pharmacy on that day; although 96.4% of prescribed medications were immediately prepared and the remaining 3.6% were ready during the day due mainly to the change of prescription amounts.

**Table 1:**
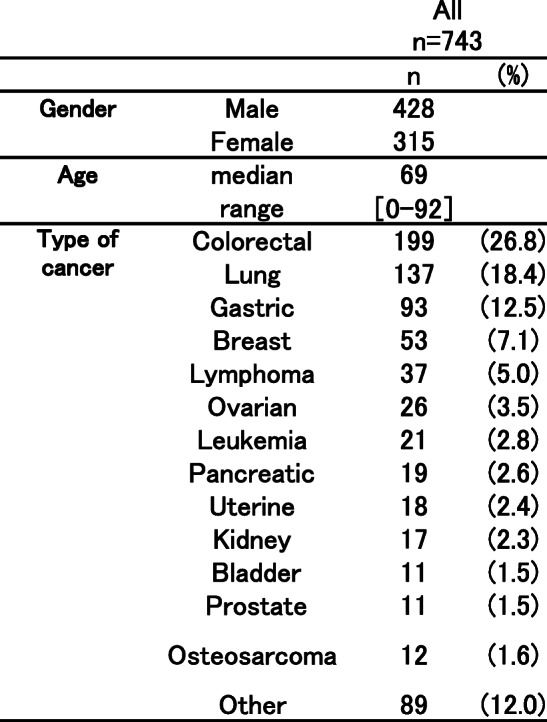
Characteristics of cancer patients with communication sheet for pharmaceutical cooperation issued by the hospital

**Fig. 3:**
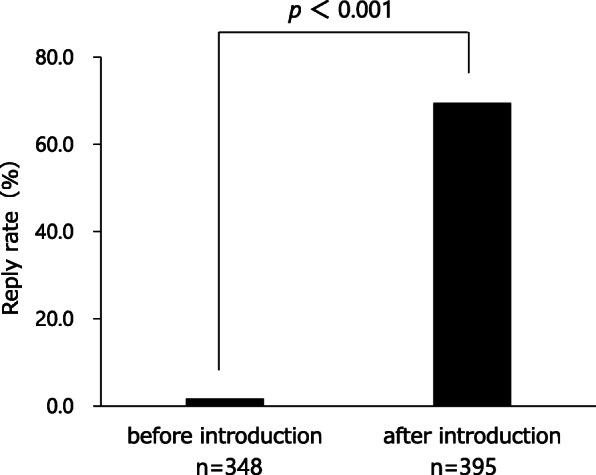
The reply rate from pharmacists was compared before and after the introduction of the reply form. Fisher’s exact test (EZR)

The results of the questionnaire survey are shown in Table 2. More than half of cancer patients still answered that they never talked with community pharmacists even 1 year after the use of the communication sheets (cancer treatment 57.7%; type of cancer 66%; administration methods of anticancer agents 77.3%; cancer treatments other than drugs 72.1%; adverse effects 71.1%). Approximately 25–45% of community pharmacists perceived that the chance of communication increased due to the use of the communication sheet. However, there were remarkable differences in the degree of favorable ratings between pharmacists and cancer patients for each item (cancer treatment: 45.4% vs 22.7%, *p* < 0.001; administration methods of anticancer agents: 41.3% vs 16.5%, *p* < 0.001; cancer treatments other than drugs: 31.0% vs 14.5%, *p* < 0.01; and adverse effects and the related treatments: 43.3% vs 22.7%, *p* < 0.001; respectively) except for the type of cancer (25.8% vs 16.5%, respectively, *p* = 0.101). Table 3 displayed the relationship between each factor (age, gender, clinical department, area of pharmacy, and oral cancer drug prescription) and incidence of communication barrier determined by univariate analysis based on the reply of question (1). The factors responsible for patients feeling difficulty talking to pharmacists about cancer therapy with *P* values < 0.20 were age and cancer drug prescription. The multivariable analysis indicated that age less than 65 years old (OR 0.301, 95% CI 0.115–0.783, *p* = 0.014) and prescriptions without an oral cancer drug (OR 0.368, 95% CI 0.154–0.879, *p* = 0.024) were independent risk factors (Table 4). Table 5 showed the relationship between each factor (age, gender, clinical department, area of pharmacy, and oral cancer drug prescription) and incidence of communication barrier determined by univariate analysis when the pharmacists talked to cancer patients. The factors that community pharmacists felt difficulty talking to patients about cancer therapy with *P* values < 0.20 were the same as cancer patients.: These factors were included in the multivariable analysis, which indicated prescriptions without an oral cancer drug (OR 0.056, 95% CI 0.007–0.459, *p* = 0.007) and age less than 65 years old (OR 0.202, 95% CI 0.064–0.639, *p* = 0.007) were independent risk factors (Table 6). To overcome these difficulties, we added a unified reply form to the communication sheet in October 2016. Thereafter, 395 external prescriptions were issued by our hospital. Among them, the number of replies returned from the community pharmacists was 274 cases as of April 30, 2019. The introduction of the unified reply form significantly increased the response rate from health insurance pharmacies (*p* < 0.001, Fig. 3). The frequency of the replies was as follows: once 256 cases; 2–5 times 12 cases; 6–9 times 2 cases; and ≧10 4 cases. According to pharmacists’ replies, both prescriptions without oral cancer drugs (OR 1.141, 95% CI 0.501–2.702, *p* = 0.848) and age < 65 years old (OR 1.189, 95% CI 0.497–3.083, *p* = 0.839) were able to be reduced with the use of the reply form for the communication sheet in Table 7.

**Table 2:**
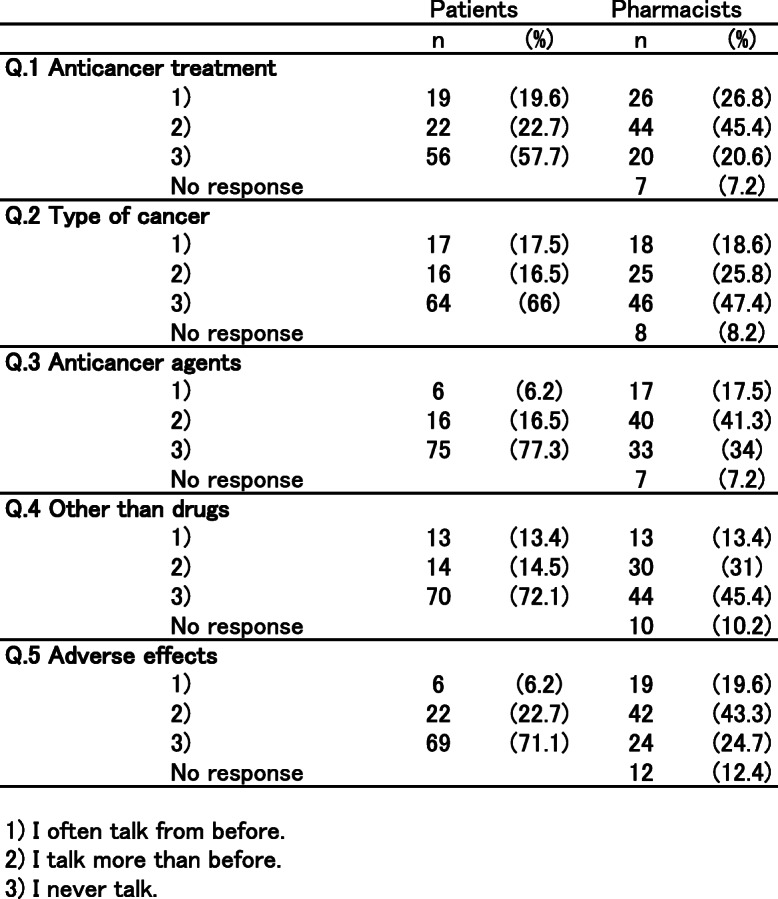
Questionnaire survey performed 1 year after the implementation of the communication sheet for pharmaceutical cooperation. 1) I often talk from before. 2) I talk more than before. 3) I never talk

**Table 3:**
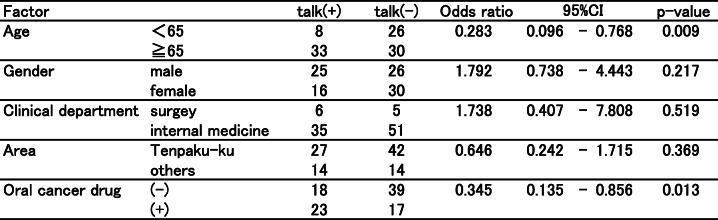
Univariate analysis of risk factors for patients feeling difficulty talking to pharmacists

**Table 4:**
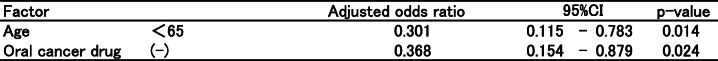
Multivariate analysis of risk factors for patients feeling difficulty talking to pharmacists

**Table 5:**
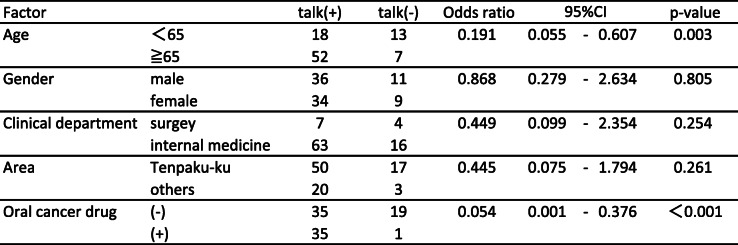
Univariate analysis of risk factors for pharmacists feeling difficulty talking to patients

**Table 6:**
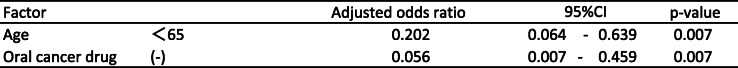
Multivariate analysis of risk factors for pharmacists feeling difficulty talking to patients

**Table 7:**
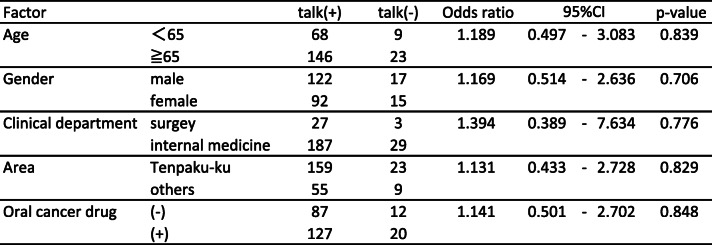
Univariate analysis of risk factors for pharmacists feeling difficulty talking to patients based on reply form

## Discussion

Currently, one-half of Japanese people have cancer, and cancer treatments are increasingly being provided in outpatient settings [19]. However, due to the lack of the stock of medications, some cancer patients with externally prescribed medications cannot receive these medications at health insurance pharmacies [17]. In addition, community pharmacists often have trouble communicating with cancer patients, mainly because they lack patients’ information, including the exact diagnosis, stage of malignant disease, treatment policy, and the status of informed consent from the patients in general [20]. We did not recognize the actual conditions of outpatient clinical pharmacy services for cancer patients until the series of discussions with community pharmacists, who felt substantial stress about how to communicate with cancer patients due to the shortage of information.

To enhance the quality of outpatient pharmacy services for cancer patients, we created a communication sheet for pharmaceutical cooperation between Nagoya Memorial Hospital and health insurance pharmacies in October 2014. By sending a communication sheet for pharmaceutical cooperation by facsimile beforehand, 96.4% of prescribed medication were immediately prepared in health insurance pharmacies on that day. Therefore, the stock of either expensive or rare medications in health insurance pharmacies, the largest challenge for our cancer patients, can be secured without any delay using the original communication sheet.

The next problem was a communication gap between community pharmacists and cancer patients. Approximately 25–45% of community pharmacists perceived that they talked to cancer patients more than before due to the use of the communication sheet (Table 2). This finding indicated that our system remarkably enhanced communication opportunities between pharmacists and cancer patients. However, there were remarkable differences in the degree of favorable ratings between pharmacists and cancer patients for each item (Table 2), suggesting that the evaluation from cancer patients was not as positive as that from pharmacists. More importantly, more than half of patients still did not talk with community pharmacists about cancer therapy and were not consulted on the adverse effects of chemotherapeutic agents (Table 2). The factors that cancer patients felt difficulty talking to pharmacists about cancer therapy were analyzed by the multivariable analysis, which indicated that age less than 65 years old (OR 0.301, 95% CI 0.115–0.783, *p* = 0.014) and prescriptions without an oral cancer drug (OR 0.368, 95% CI 0.154–0.879, *p* = 0.024) were independent risk factors (Table 4). The same tendency was observed among the community pharmacists (Table 6). These results suggested that pharmacists’ instructions at the window were remarkably affected by the route of administration of the antineoplastic agents and age of the cancer patients. To overcome these problems, we revised the communication sheet for pharmaceutical cooperation and added a unified reply form to prompt the inquiry or response from community pharmacies. This modified system enabled community pharmacists to share patients’ information with hospital staff by sending the reply form by facsimile and receiving a quick telephone reply from the hospital. To facilitate bidirectional information sharing, this process significantly decreased the difficulty in communication with cancer patients who were not prescribed oral anticancer agents and who were younger than 65 years old (Table 7).

Intimate communication and bidirectional sharing of patients’ information between hospital staff and community pharmacies are important to enhance the satisfaction of cancer patients [15, 16], although few studies have objectively assessed the satisfaction of patients receiving chemotherapeutics [2]. We collaborated with the regional dental associations [21, 22]and the facilities of palliative care and home care [23]using the information sheet to increase the quality of life in cancer patients. This multi-professional collaboration consequently led to repeated discussions with the regional pharmaceutical associations near our hospital (Tenpaku-ku, Midori-ku, and Nisshin-shi), during which we decided to create a communication sheet for pharmaceutical cooperation [17]. Based on the results of the questionnaire survey conducted after the use of the communication sheet, the original communication sheet was modified, and a unified reply form was added. We then reassessed the quality of communication between patients and pharmacists before and after revision of the communication sheet, which suggested that our system of pharmaceutical cooperation helped community pharmacists provide cancer patients with an appropriate instructions [17, 24].

## Conclusions

Bidirectional information sharing using the communication sheet enhanced communication among hospital staff members, community pharmacists, and cancer patients, which should improve cancer medication management.

## Data Availability

The datasets supporting the conclusions of this article are included within the article.

## Abbreviations

CTCAE ver. 4: Common Terminology Criteria for Adverse Events Version 4.0
OR: Odds ratio
95% CI: 95% confidence interval
